# A Quorum Sensing Small Volatile Molecule Promotes Antibiotic Tolerance in Bacteria

**DOI:** 10.1371/journal.pone.0080140

**Published:** 2013-12-19

**Authors:** Yok-Ai Que, Ronen Hazan, Benjamin Strobel, Damien Maura, Jianxin He, Meenu Kesarwani, Panagiotis Panopoulos, Amy Tsurumi, Marlyse Giddey, Julie Wilhelmy, Michael N. Mindrinos, Laurence G. Rahme

**Affiliations:** 1 Department of Surgery, Harvard Medical School and Massachusetts General Hospital, Boston, Massachusetts, United States of America; 2 Department of Microbiology and Immunobiology, Harvard Medical School, Boston, Massachusetts, United States of America; 3 Shriners Hospitals for Children Boston, Boston, Massachusetts, United States of America; 4 IYAR, The Israeli Institute for Advanced Research, Israel; 5 Stanford Genome Technology Center, Stanford University, Palo Alto, California, United States of America; 6 Institute of Dental Sciences and School of Dental Medicine, Hebrew University, Jerusalem, Israel; 7 Department of Fundamental Microbiology, University of Lausanne, Lausanne, Switzerland; The Scripps Research Institute and Sorrento Therapeutics, Inc., United States of America

## Abstract

Bacteria can be refractory to antibiotics due to a sub-population of dormant cells, called persisters that are highly tolerant to antibiotic exposure. The low frequency and transience of the antibiotic tolerant “persister” trait has complicated elucidation of the mechanism that controls antibiotic tolerance. In this study, we show that 2’ Amino-acetophenone (2-AA), a poorly studied but diagnostically important small, volatile molecule produced by the recalcitrant gram-negative human pathogen *Pseudomonas aeruginosa*, promotes antibiotic tolerance in response to quorum-sensing (QS) signaling. Our results show that 2-AA mediated persister cell accumulation occurs via alteration of the expression of genes involved in the translational capacity of the cell, including almost all ribosomal protein genes and other translation-related factors. That 2-AA promotes persisters formation also in other emerging multi-drug resistant pathogens, including the non 2-AA producer *Acinetobacter baumannii* implies that 2-AA may play an important role in the ability of gram-negative bacteria to tolerate antibiotic treatments in polymicrobial infections. Given that the synthesis, excretion and uptake of QS small molecules is a common hallmark of prokaryotes, together with the fact that the translational machinery is highly conserved, we posit that modulation of the translational capacity of the cell via QS molecules, may be a general, widely distributed mechanism that promotes antibiotic tolerance among prokaryotes.

## Introduction

Antibiotic tolerance, observed in a broad range of microbial species, is the capacity of bacterial sub-populations to tolerate exposure to normally lethal concentrations of bactericidal antibiotics [1,2]. This ability, which is not due to antibiotic-resistant mutants, has been implicated in antibiotic treatment failures [3,4] and may account for latent, chronic, and relapsing infections that can be suppressed, but not eradicated. Drugs that target such infections are lacking, and the phenomenon of antibiotic tolerance remains poorly understood. It has been suggested that antibiotic tolerant cells or “persisters” are formed as a result of stochastic events that generate phenotypic variability [5-7] in a genetically homogenous population [8]. And despite the identification of several functions involved in this process, including an SOS response, toxin-antitoxin modules, and global regulators [5,7-11], the mechanism of persister cells formation remains not fully understood.

The kinetics of persister cells accumulation implies that a form of cell-to-cell signaling known as quorum sensing (QS) may affect persisters’ formation. QS is a cell density dependent phenomenon based on the extracellular release of low molecular weight molecules that coordinate gene expression in a bacterial cell population [12-14]. While a link between cell-to-cell signaling and persister cells formation has been reported [15-17], the actual role of QS and the mode of action of QS regulated molecules in persister formation remain largely unknown. 

Studies with *P. aeruginosa* have contributed greatly to our understanding of QS signaling via the paradigmatic complex population density communication networks*. P. aeruginosa* harbors at least three QS systems that control more than 10% of its genome, allowing the pathogen to adapt to various environments and hosts [13,18]. Two of them, controlled by the regulators LasR and RhlR are induced by derivatives of Acyl-homoserine lactones. The 3^rd^ one is controlled by the multiple virulence factor regulator, MvfR [PqsR] [19]. MvfR directs the synthesis of its own ligands, hydroxy-2-heptylquinolone (HHQ) and 3,4-dihydroxy-2-heptyquinoline (PQS) [12], via a feedback mechanism involving its binding to the *pqsABCD* operon [20-22], and regulates the production of many QS regulated virulence factors and that are essential for full pathogenesis in mammals and several non-mammalian host models [12,19,23-25].

*P. aeruginosa* cultures are characterized by a sweet grape-like odor. This odor has been attributed to the small-excreted volatile molecule, 2’-amino acetophenone (2-AA), which is used to diagnose *P. aeruginosa* infections in humans [26,27]. 2-AA, along with a large number of small molecules including 4-hydroxy-2-alkylquinolines (HAQs), is synthesized by *pqsABCD* operon enzymes [12,18,28,29], which are under the transcriptional control of MvfR [12,18,28,29]. This non-HAQ molecule, is an abundant MvfR-regulated molecule that mediates phenotypic changes in a sub-population of cells which may contribute to chronic infections by stochastically silencing acute virulence functions in *P. aeruginosa* [29]. We recently shown that 2-AA also acts as an immunomodulatory signal that promotes original aspects of inter-kingdom regulation – it modulates host immune responses in a manner that increases the host’s ability to cope with this pathogen enabling host tolerance to infection and long-term bacterial presence [30]. These findings, combined with the presence of 2-AA in difficult to treat *P. aeruginosa*-infected burn wounds [27] and *P. aeruginosa* clinical isolates from cystic fibrosis patients [31], prompted us to hypothesize that 2-AA may be involved in *P. aeruginosa* antibiotic tolerance.

## Results

### 2-AA promotes accumulation of antibiotic tolerant cells in *P. aeruginosa*

**Figure 1:**
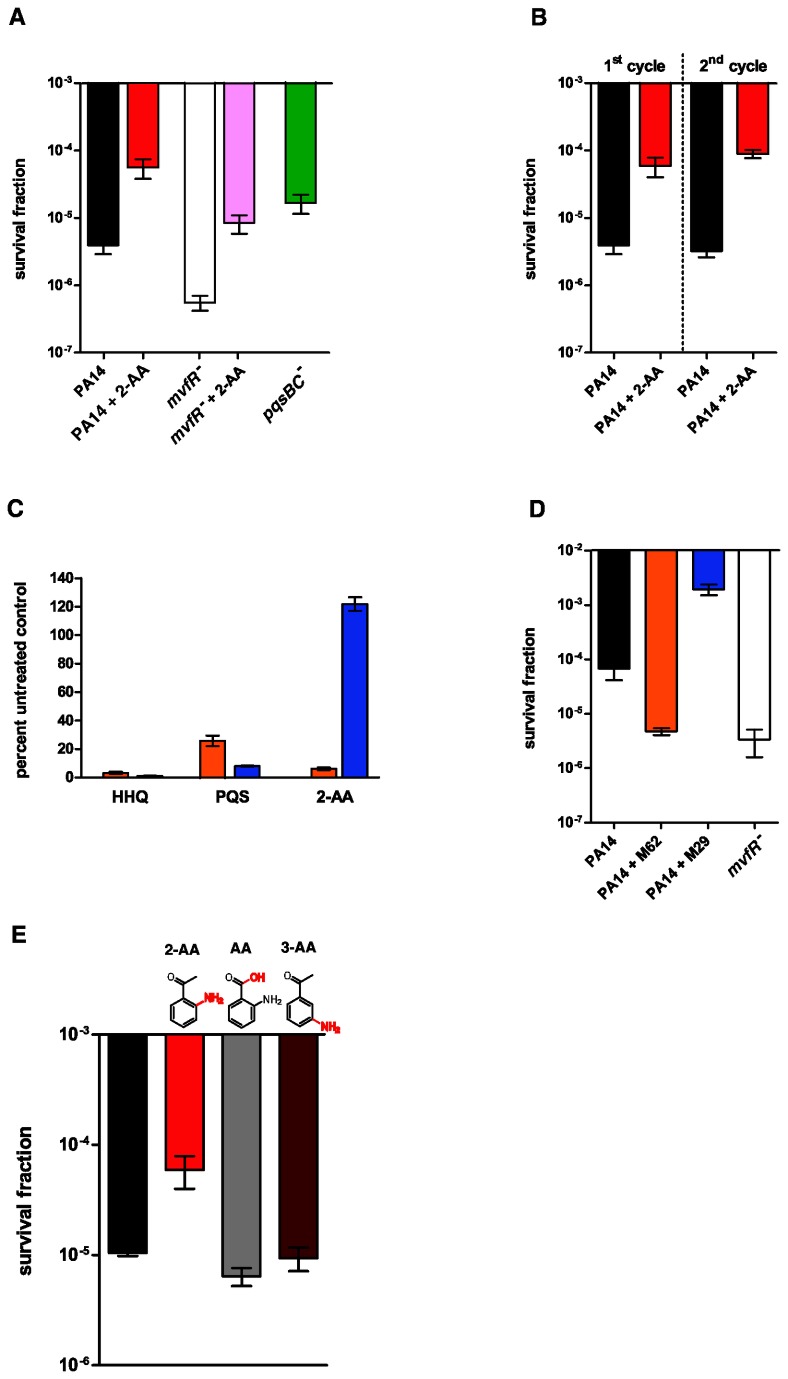
2-AA promotes antibiotic tolerance in *P. aeruginosa*. **A**. Survival fraction of controls PA14 (black), PA14 + 2-AA (red), and *mvfR* ^-^ (white), *mvfR* ^-^ + 2-AA (pink) and *pqsBC* ^-^ (green) cells grown to OD_600nm_= 2.0 followed by the addition of 10 μg/ml meropenem. All experiments were performed in triplicates, and results expressed as mean ± SD. Differences in persister fractions between PA14, *mvfR* ^-^ and pqsBC^-^ (*p*-value = 0.0016), between PA14 and PA14 + 2-AA (*p*-value = 0.0078) and between *mvfR* ^-^ and *mvfR* ^-^ + 2-AA (*p*-value = 0.0062), are statistically significant (one-way ANOVA, Tukey’s HSD test). **B**. Persister cells are antibiotic tolerant, not antibiotic resistant. After meropenem killing (1^st^ cycle), a single PA14 surviving colony was re-inoculated in fresh medium in the presence (red) or absence (black) of 2-AA (*p*-value = 0.0078, Student’s t-test), and antibiotic was applied again for 24 h (2^nd^ cycle) (*p*-value = 0.0003, Student’s t-test). Additional single colonies tested gave similar results. **C**, **D**. Chemical inhibition/stimulation of 2-AA production modulates persisters formation. Inhibition of 2-AA production (C) by compound M62 (orange) added to PA14 (black) culture decreased persisters formation (D) to a level similar to *mvfR* ^-^ mutant (white), and stimulation of 2-AA production (C) by M29 (blue) increased PA14 persisters formation (D). The experiment was performed in triplicates, and results are expressed as mean ± SD. Differences in the persisters fractions between PA14, PA14 + M62 and PA14 + M29 are statistically significant (*p*-value=0.0001, one-way ANOVA, Tukey HSD). **E**. The accumulation of persister cells is 2-AA specific, as neither the precursor/analog AA (grey) nor the analog 3-AA (brown) increased the persister cell fraction. The experiment was performed in triplicates, and results are expressed as mean ± SD. Differences in the persisters fractions are only statistically significant between PA14 (black) and PA14 + 2-AA (red) (*p*-value=0.0004, one-way ANOVA, Tukey HSD).

The synthesis of 2-AA is under the control of the QS regulator MvfR via the transcriptional regulation of the *pqsABCD* operon genes and requires *pqsA* and *pqsD* genes but not *pqsB* or *pqsC* [29]. To assess the 2-AA role on persisters formation first we used the PA14 isogenic *mvfR* ^*-*^ and *pqsBC* ^*-*^ mutants and compare them to the parental strain (Figure 1A) following exposure to high concentrations of a bactericidal antibiotic (i.e. meropenem). *mvfR* ^*-*^ cells, which do not produce 2-AA and HAQs, exhibited 10 times fewer persisters than the wild-type (WT) cells (Figure 1A), while *pqsBC* ^*-*^ that produces 2-AA but not HAQs [29], exhibited higher levels of persisters than did *mvfR* ^*-*^ mutant (Figure 1A). As also shown in Figure S1A the majority of PA14, *mvfR* ^*-*^ *and pqsBC* ^*-*^exponential-phase cells died quickly, showing a sharp but different drop-off in survival kinetics within 24 h and 48 h. Their persister fractions are comparable to previously reported persister cells rate [32-34]. Consistently, a smaller fraction of *mvfR* ^*-*^ cells compared to those of PA14 and *pqsBC* ^*-*^ survived the treatment even following 48 h exposure to high concentrations of antibiotic (Figure S1). Exogenous addition of 2-AA increased significantly (*p* < 0.05) in a dose dependent manner (Figure S1B) the surviving fraction of PA14 cells (Figure 1A), by up to 16 fold. Similarly, exogenous addition of 2-AA increased the antibiotic tolerant cell fraction in *mvfR* ^*-*^cells (Figures 1A and S1B), in which it restored persister formation in a manner toward that of WT cells, confirming that the *mvfR* ^*-*^ cell’s deficit in persister formation is likely due to 2-AA. The surviving cells were confirmed to be truly antibiotic tolerant and not resistant mutants; this tolerance characteristic was demonstrated by their unchanged minimal inhibitory concentrations (MICs) (Table S1) and by repetition of the killing curve for a culture inoculated from a single surviving colony (Figure 1B). These effects were observed without any significant alterations on either the growth rate (Figures S2A-C) or sensitivity to meropenem (Figure S2D and Table S1). It should be noted that 2-AA effect is not limited to bacteria that tolerate meropenem only but also tolerant to other antibiotic (i.e. tetracycline, Figure S3).

To further confirm the correlation between 2-AA production and persisters formation we used two novel MvfR regulon inhibitors we have identified recently (Starkey et. al submitted). The first, M62 significantly reduced the production of HHQ, PQS and 2-AA, while M29 although inhibited HHQ and PQS production, increased 2-AA levels (Figure 1C). Accordingly, while M62 significantly reduced PA14 persisters to a similar level as in *mvfR* ^*-*^, M29 increased the levels of PA14 persisters (Figure 1D). Furthermore, Figure 1E shows that promoting persister formation is specific to 2-AA, as it does not occur with 2-AA analogs such as 3-AA and anthranilic acid (AA). Taken together, these results demonstrate that the small volatile QS molecule 2-AA promotes persisters formation in *P. aeruginosa*.

### 2-AA decreases the transcription of many genes involved in the translational capacity of the cell, contributing to persister formation

**Figure 2:**
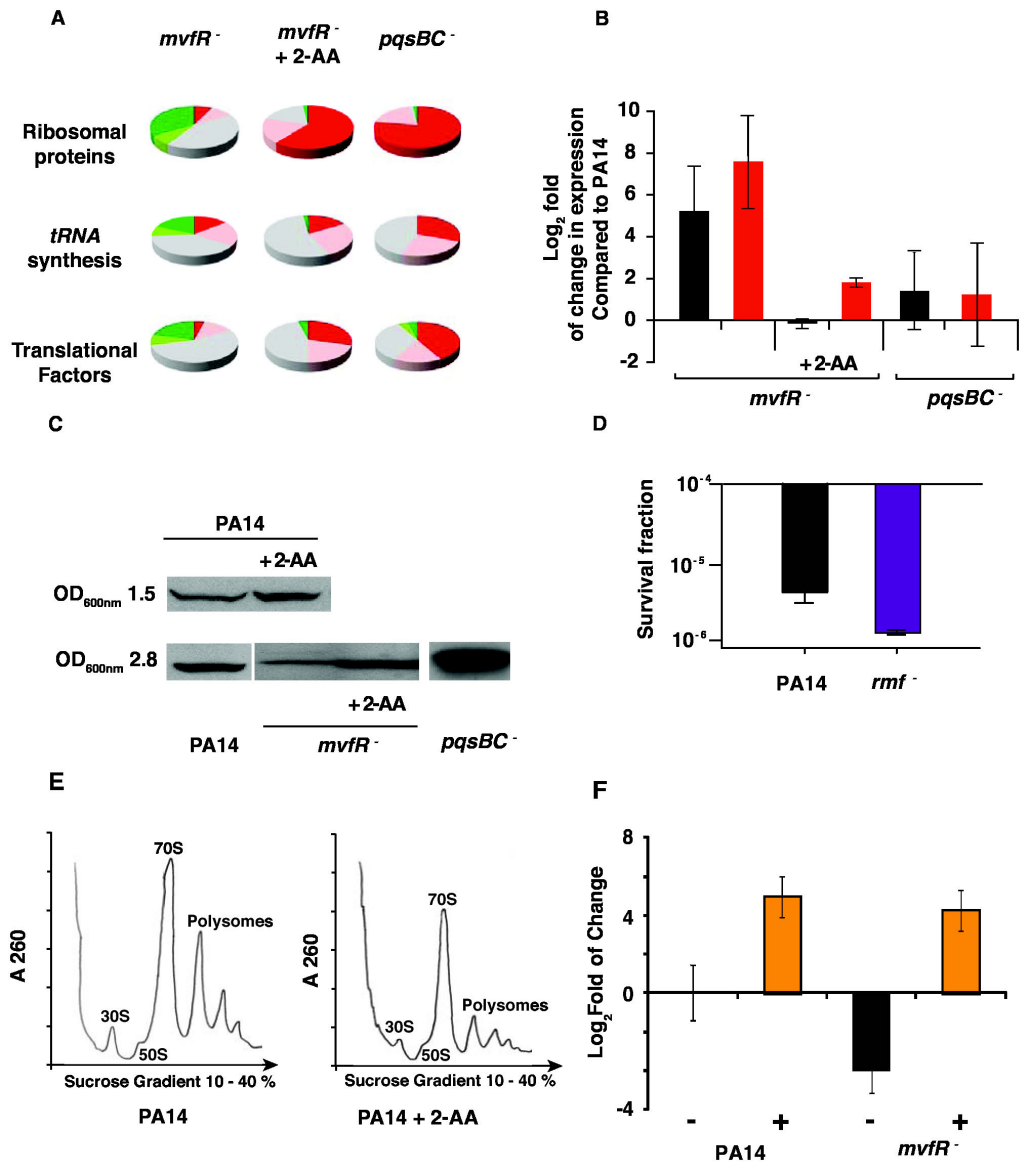
2-AA down-regulates the transcription of many genes involved in the translational capacity of the cell. **A**. Graphical analysis of *mvfR* ^-^ *, mvfR* ^-^+ 2-AA and *pqsBC* ^-^ transcriptomes to examine expression of all genes involved in translation (Table S2). Down-regulated genes are shown as red (2-fold or more reduction), or pink (1.5- to 2-fold reduction), and up regulated genes as green (>2-fold increase) or light green (1.5- to 2-fold increase). No change in gene expression is shown as grey. **B**. Expression of 16 rRNA (black) and 23S rRNA (red) in *mvfR* ^-^ and *pqsBC* ^-^cells was assessed by qRT-PCR in the absence or presence (+ 2-AA) of exogenously added 2-AA. The PA14 group +/- 2-AA was used as the calibration reference. **C**. Western blot showing Rmf levels in PA14, *mvfR* ^-^, and *pqsBC* ^-^ cells. Baseline Rmf levels was markedly lower in *mvfR* ^-^ cells than in PA14 cells; Rmf levels were increased in *pqsBC* ^-^, and in both PA14 and *mvfR* ^-^ cell types in the presence of exogenously added 2-AA. **D**. *rmf* ^--^ cells (purple) exhibit a reduced persister fraction compared to PA14 cells (black). Differences between both strains are statistically significant (*p*-value = 0.0113, Student’s t-test). Experiment was performed in triplicate. **E**. Exogenously added 2-AA decreases polysomes levels. Ribosomal extracts from PA14 cells grown in the absence (left panel) or presence (right panel) of exogenously added 2-AA were separated in a 25% to 5% sucrose density gradient. The ratio of translating polysomes to 70S was higher in the untreated cultures (left) than in the cultures grown in presence of exogenously added 2-AA (right) reflecting a decrease in translational activity. **F**. Inhibition of PA14 translational capacity increase persisters formation. PA14 and *mvfR* ^-^ cells were grown in absence (black) or presence (orange) of sub-MIC concentration of translational inhibiting antibiotic chloramphenicol (15 mg/L), which did not affect PA14 growth (data not shown). Persisters fraction was determined as described in Figure 1. The results are presented as log_2_ fold of change of the untreated PA14 persisters fraction as mean ± SD.

Previous studies in *E. coli* have shown that translation arrest is involved in dormancy and persister formation [11,35-37]. A series of findings, as presented in Figure 2, support our deduction that the 2-AA impacts the translational capacity of the cell. First, the transcription of almost all ribosomal protein genes, most *tRNA* synthesis genes, and numerous translational factors involved in translation initiation, elongation, and release—including EF-Tu, which has been hypothesized to be involved in persister formation [11] is significantly down-regulated in 2-AA treated *mvfR* ^-^ and *pqsBC* ^-^compared to *mvfR* ^-^ (Table S2 and Figure 2A). Second, expression of the aforementioned translation-related genes, including that of 16S and 23S *rRNA* (Figure 2B), is significantly up regulated in *mvfR* ^-^ transcriptome profiles compared to PA14, (Figures 2A-B and Table S2). Third, gene expression (Table S2) and protein levels (Figure 2C) of the ribosomal modulation factor (RMF), which is known to promote ribosomal inactivity [38,39], was increased by 12.65-fold following addition of 2-AA and by 15.9 fold in *pqsBC* ^-^ cells (Table S2). Moreover, as shown in Figure 2C, RMF protein levels in PA14 cells were prematurely increased at OD_600nm_ 1.5 following exogenous addition of 2-AA, and the low RMF protein levels in *mvfR* ^-^ cells increased at the late exponential phase (OD_600nm_ 2.8) when 2-AA was added. Accordingly, loss of RMF function leads to a decreased persister fraction of about 5 fold compared to the parental strain (Figure 2D). Moreover, the increased *rmf* expression observed in *pqsBC* ^-^ cells (Figure 2C) is in agreement with their increased persister fractions (Figures 1A and S1). Fourth, relative to non-2-AA-treated PA14 cells, the overall translational capacity of 2-AA treated PA14 cells was found to be decreased, as reflected by the low ratio between the translating polysomes and the 70S subunit (Figure 2E). Lastly, as shown in Figure 2F modulation of the cell translation capacity that does not affect growth impacted persisters formation. Addition of sub-MIC concentration of the translation inhibitor chloramphenicol increased persisters levels by 16-64-fold in both PA14 and *mvfR* ^-^ mutant cells (Figure 2F).

**Figure 3:**
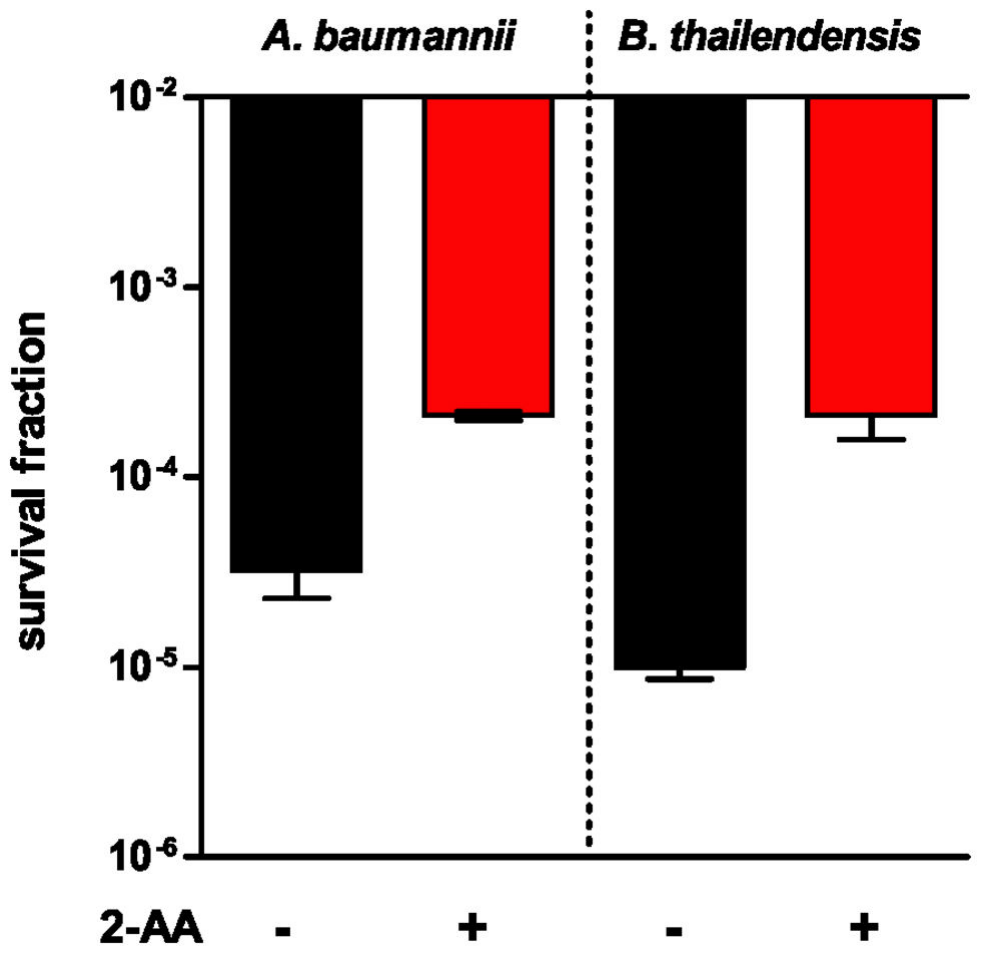
2-AA increase persister cell formation in other bacteria. Survival fraction of *Acinetobacter baumannii* (left panel), and *Burkholderia thailandensis* (right panel) cells grown to OD_600nm_= 2.0 in the absence (black) or presence (red) of 2-AA followed by the addition of 10 μg/ml meropenem. All experiments were performed in triplicates, and results expressed as mean ± SD. Differences in persister fractions between absence and presence of 2-AA are statistically significant for both *A. baumannii* (*p*-value = 0.0068, Student’s t-test) and *B. thailandensis* (*p*-value = 0.0203, Student’s t-test).

These results provide strong support for the notion that the 2-AA mediated transcriptional modulation alters the translation capacity of the cell, and thus promotes persister cell accumulation. Taking into consideration the conserved nature of the translational machinery, we hypothesized that 2-AA may also promote persisters formation in other bacteria. Indeed, Figure 3 shows that 2-AA increased persisters accumulation in other two pathogens, *Burkholderia thailandensis* and *Acinetobacter baumannii* that are frequently isolated together with *P. aeruginosa* [40,41]. These data suggest that 2-AA may promote antibiotic tolerance in polymicrobial communities across bacterial genera.

## Discussion

It is well documented that QS is mediated by diffusible small molecules that serve as intricate signals to exert transcriptional control over a vast array of genes, many of which encode virulence factors [42]. The presently reported group of experiments provides a direct link among the QS volatile molecule 2-AA and antibiotic tolerance, persister cells, through the modulation of the translational capacity of the cell. Given that the synthesis and excretion of small molecules is a common hallmark of prokaryotes, including many for which QS has been described, together with the facts that the translational machinery is highly conserved among bacterial species and that 2-AA is a widespread molecule, found in both complex and primitive organisms, we posit that the effect of 2-AA to promote antibiotic tolerance in prokaryotes, may be widely distributed as suggested by its impact on *Burkholderia* and *Acinetobacter* and that may play a crucial role in antibiotic tolerance regulation in polymicrobial communities. 

Interestingly, while many *P. aeruginosa* virulence factors associated with acute infections are controlled by MvfR, 2-AA, which is an MvfR natural “inhibitor” and regulated molecule, prolongs bacterial survival and mediates changes in a subpopulation of cells that facilitate the exploitation of dynamic host environments while promoting gene expression changes that favor chronic infections [29,31]. In evolutionary terms, persisters, being a small fraction of the cell population, could be considered as part of the cell cycle “life insurance policy”, allowing a few bacterial cells to survive unpredictable environmental stresses and thus secure long-term survival. *Bacillus* spores represent an extreme example of such a “life insurance policy” [43]. Many bacterial secreted natural small molecules are currently thought to be antibiotic “weapons” aimed at controlling competitors. We propose that such small excreted molecules, at sub-MIC concentrations, could also be promoting bacterial persistence within bacterial communities by preparing some cells to survive unpredictable stresses.

Our results show that 2-AA promotes phenotypic diversity by promoting persister cell formation via transcriptional modulation of the translational capacity of the cell. Translation arrest has been shown to be involved in bacterial cell dormancy and persister formation in *E. coli* by experiments examining RelA [35], the enzyme responsible for the synthesis of guanosine 3’, 5’- bis (diphosphate) penta phosphate (ppGpp) [36,37,44], and experiments examining HipA, a protein kinase and component of a toxin-antitoxin module that phosphorylates the essential translation factor EF-Tu [11]. There is no *hipA* homolog in *P. aeruginosa* to be tested. However, 2-AA does alter EF-Tu gene and protein expression in *P. aeruginosa*, without altering *relA* expression (GEO, # GSE24036), which is not surprising since *relA* is known to be mainly regulated at the protein level [45]. The involvement of RMF further emphasizes the link between translation arrest and persisters formation, as RMF accumulation causes sequestration and inactivation of ribosomal subunits, decreasing the translational capacity of the cell [38,39]. Indeed, RMF has previously been implicated in *E. coli* persister formation [46]. Moreover, RMF contributes to heightened levels of gene expression noise during stationary phase, suggesting that it could provide phenotypic diversity under adverse conditions [47]. This possibility is supported by our observations that PA14 *rmf* ^–^ cells form fewer persisters than their parent PA14 cells (Figures 2D). These results also suggest that persister accumulation may be promoted in *P. aeruginosa* by alternative components to 2-AA that ultimately affect the same cellular functions, such as regulators of translation. 

Our previous study [29] suggested that 2-AA may promote phenotypic heterogeneity in a genetically “homogenous” population in a stochastic manner acting as a typical phenotypic switch characteristic of persister cells [6]. Interestingly, 2-AA effects are promoted by negatively regulating the MvfR regulon and as a result the production of HAQs, suggesting antagonism between HAQs and 2-AA. 

2-AA impacts the transcription of translation-related genes, thereby affecting the cell’s translational capacity, resulting in the formation of persisters. Intrinsic molecules, or those produced by the surrounding microbiota, may promote such modulation in natural microbial communities. Our data support the notions that persister formation may be a subpopulation response to cell density, and that such “phenotypic noise” [6], which is beneficial for the long-term presence of the microbe, is mediated through QS. All experiments were conducted on planktonic cultures. Whether 2-AA also promotes persister cell formation in biofilms and it is involved in biofilm-related tolerance remains unknown. 

QS components are currently among the most investigated targets in the ongoing battle against antibiotic resistance. The tight link between MvfR-QS system and persister cell formation highlights the importance of MvfR as a highly promising target for the development of inhibitors that can simultaneously halt acute virulence and the antibiotic tolerance that leads to chronic and relapsing infections. Moreover, the immunomodulatory capacity of the MvfR-regulated molecule 2-AA that facilitates pathogen persistence, while enabling host tolerance to infection, and our more recent findings showing that 2-AA causes further harm to the host by triggering mitochondrial dysfunction in skeletal muscle [48], further highlights the importance of inhibiting the MvfR-QS system. Normal skeletal muscle function is essential to survival, and is compromised in many chronic illnesses, including infections and CF-associated muscle wasting. Overall, our findings reveal new insights into the mechanisms of antibiotic tolerance and open new avenues for the development of novel antibiotic tolerance inhibitors that will not only prevent bacterial virulence but also immunomodulatory signals that promote bacterial long-term presence and mitochondrial dysfunction in mammals. The development of such treatments is of the utmost importance given that persistent infections are highly prevalent, difficult to eradicate, and often untreatable, posing grave threats to human health worldwide. 

## Methods

### Strains, antibiotics, and chemicals

Unless otherwise stated, bacteria were grown overnight at 37 °C in LB (Difco, Detroit) with aeration, or on LB agar plates containing the called for antibiotic or compounds. The *pqsBC* ^*-*^mutant construction procedure is described elsewhere [29]. *Acinetobacter baumannii* is a clinical isolate (Shriners Hospitals for Children Boston). *Burkholderia thailandensis* is closely related to *Burkholderia pseudomallei* [49]. AA, 2-AA, 3-AA and tetracycline were obtained from Sigma-Aldrich (St. Louis, MO), Meropenem from Astra-Zeneca (Newark, DE). Compounds were added at concentration of 0.75mM except for Figure 3 where 1.5mM was used for *Burkholderia*. M29 and M62 compounds are described in (Starkey et al. submitted).

### Persisters assessment

Persisters were assessed by using the standard plating method [50] and the new Start of Growth Time (SGT) method recently described by our group [51], where over-night cultures were diluted 1:100 in triplicate in fresh LB medium in the presence or absence of 2-AA, and re-grown to an OD_600nm_ of 2.0 while shaking under aeration at 37°C. This took approximately 4 hours. Before the antibiotic treatment, aliquots were plated for reference colony counts. The cultures were then treated with 10 mg/L of meropenem or tetracycline and further incubated at 37°C while shaking under aeration. At 24 h, the number of surviving bacteria was assessed by plating. The survival fraction (persisters) was calculated by dividing the number of surviving bacteria by the number of live bacteria that were present before the antibiotic treatment and expressed as a mean ± SD of at least three replicate experiments. 

### Statistical analysis

Growth curves were compared by permutation tests using the Thompson and Smyth method (http://bioinf.wehi.edu.au/software/compareCurves/) [52,53] with 10,000 permutations. Killing curves were compared by applying a one step decay exponential model (GraphPad Prism 5.04, Graphpad Software, Inc.). Persister fractions were compared using Student’s t-test or Analysis of Variance (ANOVA) and Tukey’s Honest Significant Difference (HSD) as indicated. Statistical significance was set at a *p* value of 0.05. 

### RNA isolation and transcriptome data generation and analysis

Transcriptome data of *mvfR* ^*-*^, *mvfR* ^-^ supplemented with 2-AA (*mvfR* ^-^ + 2-AA) and *pqsBC* ^*-*^ were generated as previously described [13]. These experiments were performed independently in triplicate. Normalized expression values were analyzed with the Bioconductor limma package and R functions (http://www.bioconductor.org/) [54]. The fold change relative to WT sample was log_2_ transformed before the sample-sample correlation coefficients were calculated. Pearson correlation coefficients of log_2_ transformed fold changes between samples were calculated with the R function “cor()” in the base “stats” package (http://www.bioconductor.org/) [54]. The transcriptome results of genes known to be involved in persister cells formation were validated by RT-PCR and results are expressed as log_2_ fold of change of 2-AA treated versus untreated cells: i) induced gene *rmf* (PA3049) (3.8 +/- 0.2); ii) repressed genes *lexA* (PA3007) (-0.8 +/- 0.2), *recA* (PA3617) (-2.1 +/- 0.4) and *sulA* (PA3008) (-1.2 +/-0.1). The microarray data have been deposited in the NCBI Gene Expression Omnibus under GEO Series accession number GSE24036 and can be accessed using the following link:

**http://www.ncbi.nlm.nih.gov/geo/query/acc.cgi?token=vzcrpuuwkwcymxu&acc=GSE24036**

### Western Blotting

Cells grown to the desired OD_600nm_ at 37°C while shaking, in presence or absence of 2-AA, were harvested by centrifugation, re-suspended in 500 µl of Bug Buster protein extraction reagent (Novagen) and lysed in presence of 10 mg/L lysozyme according to the manufacturer’s instructions at room temperature by gentle inversion for 30 min. After removal of cellular debris by centrifugation, the Bradford method was used to calculate protein concentrations according to the manufacturer’s protocol (Thermo Scientific Product #23238). Equivalent amounts of total proteins (5 µg) were heated for 5 min at 95°C in Laemmli sample buffer (Biorad), separated by SDS-PAGE on 12% pre-cast polyacrylamide gels (Biorad), and transferred to PVDF membranes (Millipore). The membranes were blocked with an excess of non-fat milk and then probed with RMF rabbit antiserum [1:2,000] in TBS-Tween. RMF bands were revealed by incubating the membranes with horseradish peroxidase-labeled goat anti-rabbit secondary antibodies [1:20,000] and TMB One Solution (Promega), according to the manufacturer’s instructions. Intensities of RMF bands within a pre-defined area were quantified using ImageJ 1.42q software (NIH, http://rsb.info.nih.gov/ij).

### Antibiotic sensitivity profile

The possible effect of 2-AA addition on antibiotic susceptibility of PA14 cells was evaluated using the following two methods

a): Broth macrodilution method

MICs of antibiotics were determined using a previously described broth macrodilution method [55] and 10^5^ CFU/mL as the starting inoculum. The MIC was defined as the lowest antibiotic concentration able to inhibit visible bacterial growth after 24 h of incubation at 37 °C.

b): Population analysis profiles

PA14 cells sensitivity to meropenem in the presence or absence of exogenously added 2-AA in various concentrations was determined by plating high number of cells (up to 10^9^ CFU/ml) onto LB plates containing 2-fold serial dilutions of antibiotics as described [56]. Population analysis profile curves were generated by plotting the numbers of colonies growing on the plates against the concentrations of antibiotic present on each plate.

### Ribosomal profiles

PA14 cells were harvested from logarithmic (OD_600nm_ 2) cultures grown in the presence or absence of 2-AA exogenously added to 1 ml of a 20 mM Tris-HCL (pH 7.4) buffer supplemented with 15 mM MgCl_2_, 30 mM NH_4_Cl_2_, and 125 mg/L chloramphenicol. The cells were lysed in the presence of 10 g/L lysozyme by three freeze/thaw cycles in liquid nitrogen. The lysed solution was subsequently treated with 10% sodium deoxycholate and 4 µl of a 1 mg/L DNaseI solution. Cellular debris was spun down and the supernatants solutions were collected. Ribosomes were subjected to centrifugation on 5–20% linear sucrose density gradients. After ultra-centrifugation in a SW40 Ti rotor (Beckman) at 285,000 ×*g* for 80 min at 4°C, gradients were fractionated by upward displacement with 70% (wt/vol) sucrose, and absorbance at 260 nm was monitored continuously by using an ISCO UA-6 UV monitor. 

## Supporting Information

Figure S1 **Biphasic killing curves of PA14, *mvfR*^-^ and *pqsBC*^-^.** **A**. PA14 (black dots), *mvfR* ^-^ (white triangles) and *pqsBC* ^-^ (green diamonds) cultures were treated with antibiotic for 48h and samples were collected at 0, 24 and 48h to assess the cells surviving fraction. The first 24h shows the fast killing of the non-persister exponential-phase cells that reached a killing plateau between 24- 48h. Antibiotic tolerant persister cells only survive the killing by antibiotic. Differences in persister fractions between PA14, *mvfR* ^-^ and *pqsBC* ^-^ are statistically significant (*p*-value = 0.0045 one-way ANOVA, Tukey’s HSD test). **B**. 2-AA increased the persister subpopulation in PA14 (black) and *mvfR* ^-^ (red) in a dose-dependent manner. Persister cells fractions were measured using the SGT method, and expressed in log_2_ fold change, using PA14 as a calibrator.
(PDF)

Figure S2 **Growth curves and antibiotic sensitivity profiling.** **A**-**B**. 2-AA does not affect the growth rates of *P. aeruginosa* cultures. PA14 (A) or *mvfR* ^*-*^ (B) cultures were grown in absence (black) or presence (red) of 2-AA and viable counts were determined after various incubation times. Experiments were carried out in triplicates and results are expressed as mean ± SD. **C**. Differences in *mvfR* ^-^ and *pqsBC* ^-^ persister fractions is not due to a trivial difference in growth rates. Growth curves of *mvfR* ^-^ (triangles) and *pqsBC* ^-^ (diamonds) cultures do not show any significant differences. Results were obtained and described as in A and B. **D**. 2-AA does not alter the sensitivity profile of *P. aeruginosa* to meropenem. Population analysis profiles of PA14 cultures in the presence (red) or absence (black) of 2-AA. High bacterial inocula (~10^9^ CFU) were serially diluted and spread on agar plates containing increasing concentrations of meropenem. Population analysis profile curves were generated by plotting the numbers of colonies growing on the plates against the concentrations of antibiotic present on each plate.
(PDF)

Figure S3 **Persister cells induced upon 2-AA addition are also tolerant to tetracycline.** Survival fraction of PA14 cells grown to OD_600nm_= 2.0 in the absence (black) or presence (red) of 2-AA followed by the addition of 150 μg/ml tetracycline. Experiment was performed in triplicates, and results are expressed as mean ± SD. Differences in persister cell fractions between 2-AA treated and not treated are statistically significant (*p* value <0.01, t-test unpaired).
(PDF)

Table S1 **2-AA does not impact MIC even when used at high concentrations.** MICs (mg/L) of meropenem.
(DOCX)

Table S2 **2-AA decrease the transcription of most of the genes belongs to the translational machinery.** Expression of translation related genes (fold changes) in *mvfR* ^-^ and *pqsBC* ^-^ relative to PA14 cells and*mvfR* ^-^ treated with 2-AA relative to *mvfR* ^-^ (*mvfR* ^-^ + 2-AA). Values <-2 are in red fonts and those >2 are in green fonts.
(DOCX)
