# Supplementary figures and images for: A Quorum Sensing Small Volatile Molecule Promotes Antibiotic Tolerance in Bacteria
Source: PLoS One. 2013 Dec 19;8(12):e80140. doi: 10.1371/journal.pone.0080140 (PMC3868577; doi:10.1371/journal.pone.0080140)

Figure S1

A

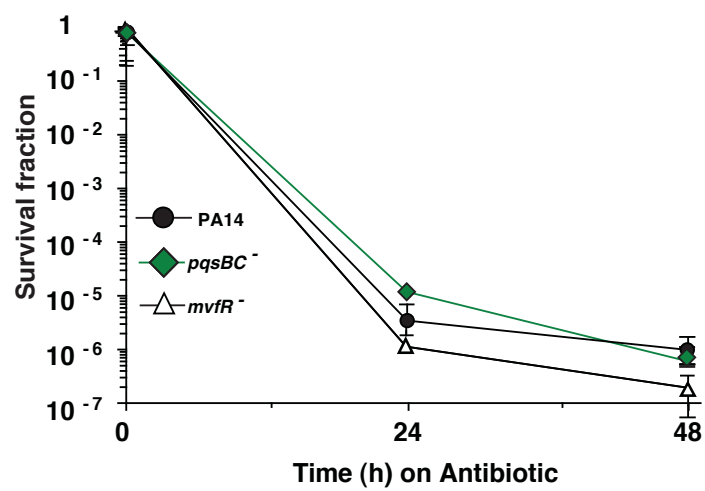

B

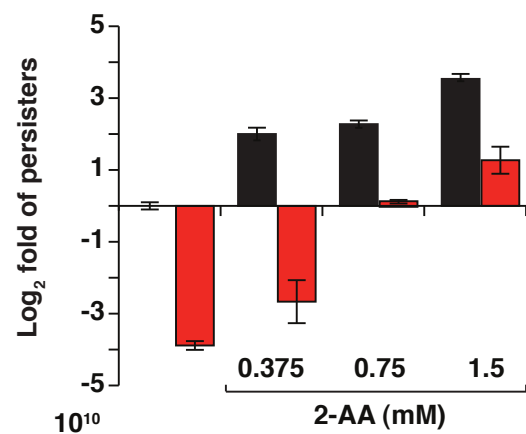

Supplement: Figure S1 — Biphasic killing curves of PA14, mvfR- and pqsBC-. A. PA14 (black dots), mvfR - (white triangles) and pqsBC - (green diamonds) cultures were treated with antibiotic for 48h and samples were collected at 0, 24 and 48h to assess the cells surviving fraction. The first 24h shows the fast killing of the non-persister exponential-phase cells that reached a killing plateau between 24- 48h. Antibiotic tolerant persister cells only survive the killing by antibiotic. Differences in persister fractions between PA14, mvfR - and pqsBC - are statistically significant (p-value = 0.0045 one-way ANOVA, Tukey’s HSD test). B. 2-AA increased the persister subpopulation in PA14 (black) and mvfR - (red) in a dose-dependent manner. Persister cells fractions were measured using the SGT method, and expressed in log2 fold change, using PA14 as a calibrator. (PDF) [file pone.0080140.s001.pdf]

**A**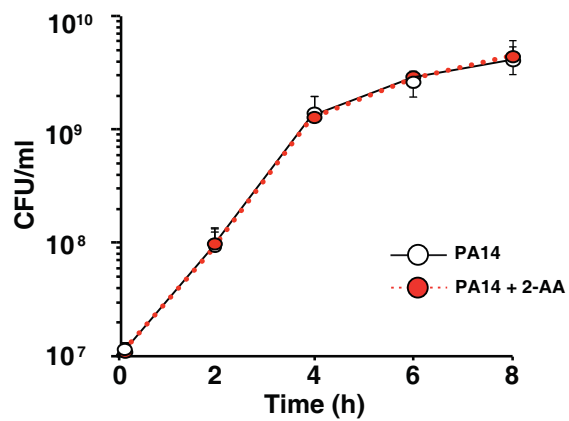**B**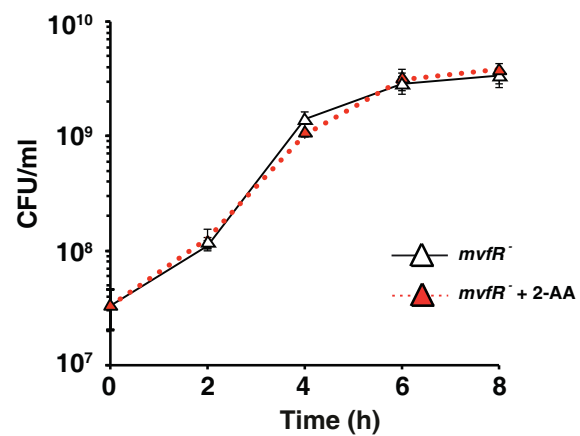**C**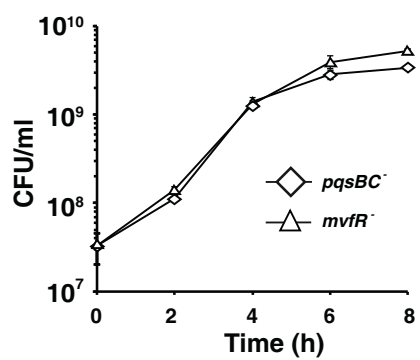**D**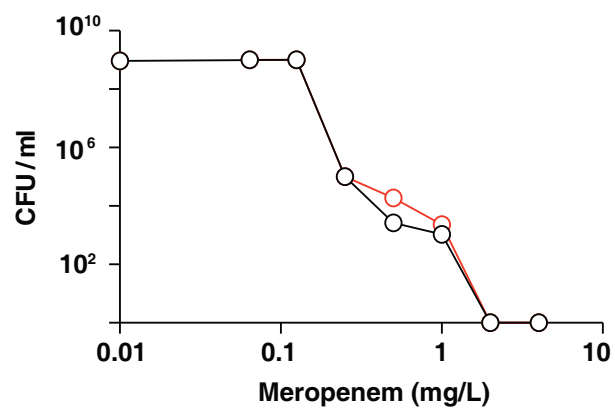**Figure S2**

Supplement: Figure S2 — Growth curves and antibiotic sensitivity profiling. A-B. 2-AA does not affect the growth rates of P. aeruginosa cultures. PA14 (A) or mvfR - (B) cultures were grown in absence (black) or presence (red) of 2-AA and viable counts were determined after various incubation times. Experiments were carried out in triplicates and results are expressed as mean ± SD. C. Differences in mvfR - and pqsBC - persister fractions is not due to a trivial difference in growth rates. Growth curves of mvfR - (triangles) and pqsBC - (diamonds) cultures do not show any significant differences. Results were obtained and described as in A and B. D. 2-AA does not alter the sensitivity profile of P. aeruginosa to meropenem. Population analysis profiles of PA14 cultures in the presence (red) or absence (black) of 2-AA. High bacterial inocula (~109 CFU) were serially diluted and spread on agar plates containing increasing concentrations of meropenem. Population analysis profile curves were generated by plotting the numbers of colonies growing on the plates against the concentrations of antibiotic present on each plate. (PDF) [file pone.0080140.s002.pdf]

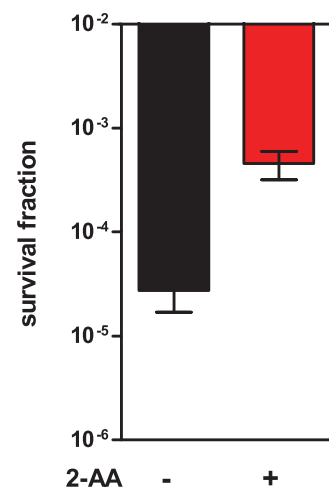

Supplement: Figure S3 — Persister cells induced upon 2-AA addition are also tolerant to tetracycline. Survival fraction of PA14 cells grown to OD600nm= 2.0 in the absence (black) or presence (red) of 2-AA followed by the addition of 150 μg/ml tetracycline. Experiment was performed in triplicates, and results are expressed as mean ± SD. Differences in persister cell fractions between 2-AA treated and not treated are statistically significant (p value <0.01, t-test unpaired). (PDF) [file pone.0080140.s003.pdf]
