# Supplementary material for: A Quorum Sensing Small Volatile Molecule Promotes Antibiotic Tolerance in Bacteria
Source: PLoS One. 2013 Dec 19;8(12):e80140. doi: 10.1371/journal.pone.0080140 (PMC3868577; doi:10.1371/journal.pone.0080140)
Supplement: Table S1 — 2-AA does not impact MIC even when used at high concentrations. MICs (mg/L) of meropenem. (DOCX) [file pone.0080140.s004.docx]

**SUPPLEMENTAL TABLE**

**Table S1**: **2-AA does not impact MIC even when used at high concentrations.**

MICs (mg/L) of meropenem.

| **Antibiotic** | **PA14** | ***mvfR^-^*** | ***pqsBC^-^*** |
| --- | --- | --- | --- |
| meropenem | 0.25 | 0.25 | 0.25 |
| meropenem + 1.5 mM 2-AA | 0.25 | 0.25 | 0.25 |
| meropenem + 3 mM 2-AA | 0.25 | 0.25 | 0.25 |
