# Supplementary material for: A Quorum Sensing Small Volatile Molecule Promotes Antibiotic Tolerance in Bacteria
Source: PLoS One. 2013 Dec 19;8(12):e80140. doi: 10.1371/journal.pone.0080140 (PMC3868577; doi:10.1371/journal.pone.0080140)
Supplement: Table S2 — 2-AA decrease the transcription of most of the genes belongs to the translational machinery. Expression of translation related genes (fold changes) in mvfR - and pqsBC - relative to PA14 cells andmvfR - treated with 2-AA relative to mvfR - (mvfR - + 2-AA). Values <-2 are in red fonts and those >2 are in green fonts. (DOCX) [file pone.0080140.s005.docx]

**SUPPORTING INFORMATION**

**Table S2:** 2-AA decrease the transcription of most of the genes belongs to the translational machinery. Expression of translation related genes (fold changes) in *mvfR*^-^ and *pqsBC*^-^ relative to PA14 cells and*mvfR*^-^ treated with 2-AA relative to *mvfR*^-^ (*mvfR*^-^ + 2-AA). Values <-2 are in red fonts and those >2 are in green fonts.

|  | **PA14 ID** | **Gene** | **PAO1 ID** | **Start** | **Stop** | ***mvfR^-^*** | ***mvfR^-^ + 2-AA*** | ***pqsBC^-^*** | | **Description** | |  |
| --- | --- | --- | --- | --- | --- | --- | --- | --- | --- | --- | --- | --- |
| **30S ribosomal proteins** | | |  |  |  |  |  |  |  |  |  |  |
|  | PA14_23330 | ***rpsA*** | PA3162_rpsA_at | 2024157 | 2025836 | -1.103 | -1.548 | **-3.146** | | 30S ribosomal protein S1 | |  |
|  | PA14_17060 | ***rpsB*** | PA3656_rpsB_at | 754693 | 755064 | 1.726 | **-2.722** | **-5.612** | | 30S ribosomal protein S2 | |  |
|  | PA14_08910 | ***rpsC*** | PA4257_rpsC_at | 5597582 | 5597851 | -1.234 | -1.354 | **-3.034** | | 30S ribosomal protein S3 | |  |
|  | PA14_09100 | ***rpsD*** | PA4239_rpsD_at | 1361045 | 1361296 | **-2.391** | -1.548 | **-3.53** | | 30S ribosomal protein S4 | |  |
|  | PA14_09020 | ***rpsE*** | PA4246_rpsE_at | 763894 | 764160 | 1.206 | -1.522 | **-5.25** | | 30S ribosomal protein S5 | |  |
|  | PA14_65180 | ***rpsF*** | PA4935_rpsF_at | 5808435 | 5808665 | **2.905** | -1.592 | **-2.972** | | 30S ribosomal protein S6 | |  |
|  | PA14_08810 | ***rpsG*** | PA4267_rpsG_at | 761956 | 762231 | 1.15 | -1.122 | -1.821 | | 30S ribosomal protein S7 | |  |
|  | PA14_08990 | ***rpsH*** | PA4249_rpsH_at | 5385127 | 5385402 | **2.62** | -1.419 | **-2.345** | | 30S ribosomal protein S8 | |  |
|  | PA14_57580 | ***rpsI*** | PA4432_rpsI_at | 651720 | 651935 | **2.766** | -1.199 | -1.246 | | 30S ribosomal protein S9 | |  |
|  | PA14_08840 | ***rpsJ*** | PA4264_rpsJ_at | 1463112 | 1463852 | **2.09** | -1.723 | **-2.184** | | 30S ribosomal protein S10 | |  |
|  | PA14_09090 | ***rpsK*** | PA4240_rpsK_at | 762589 | 763275 | -1.893 | -1.844 | **-3.762** | | 30S ribosomal protein S11 | |  |
|  | PA14_08790 | ***rpsL*** | PA4268_rpsL_at | 770752 | 771372 | -1.31 | -1.195 | **-3.637** | | 30S ribosomal protein S12 | |  |
|  | PA14_09080 | ***rpsM*** | PA4241_rpsM_at | 767249 | 767749 | -1.691 | -1.782 | **-5.447** | | 30S ribosomal protein S13 | |  |
|  | PA14_08980 | ***rpsN*** | PA4250_rpsN_at | 5808695 | 5809114 | -1.403 | -1.305 | -1.398 | | 30S ribosomal protein S14 | |  |
|  | PA14_62720 | ***rpsO*** | PA4741_rpsO_at | 755164 | 755634 | 1.744 | -1.011 | **-2.734** | | 30S ribosomal protein S15 | |  |
|  | PA14_15970 | ***rpsP*** | PA3745_rpsP_at | 765947 | 766339 | **2.052** | 1.136 | **-4.172** | | 30S ribosomal protein S16 | |  |
|  | PA14_08940 | ***rpsQ*** | PA4254_rpsQ_at | 5128667 | 5129059 | -1.719 | -1.841 | **-4.934** | | 30S ribosomal protein S17 | |  |
|  | PA14_65170 | ***rpsR*** | PA4934_rpsR_at | 759165 | 759476 | **3.247** | -1.644 | **-2.418** | | 30S ribosomal protein S18 | |  |
|  | PA14_08890 | ***rpsS*** | PA4259_rpsS_at | 770346 | 770735 | -1.21 | -1.811 | **-2.437** | | 30S ribosomal protein S19 | |  |
|  | PA14_60400 | ***rpsT*** | PA4563_rpsT_at | 769971 | 770327 | **3.611** | 1.357 | 1.009 | | 30S ribosomal protein S20 | |  |
|  | PA14_07560 | ***rpsU*** | PA0579_rpsU_at | 765452 | 765757 | **4.042** | **2.334** | -1.867 | | 30S ribosomal protein S21 | |  |
| **50S ribosomal proteins** | | |  |  |  |  |  |  | | |  | |
|  | PA14_08730 | ***rplA*** | PA4273_rplA_at | 744140 | 744835 | 1.057 | -1.750 | **-3.8** | | 50S ribosomal protein L1 | |  |
|  | PA14_08880 | ***rplB*** | PA4260_rplB_at | 5129074 | 5129502 | 1.13 | -1.430 | **-2.366** | | 50S ribosomal protein L2 | |  |
|  | PA14_08850 | ***rplC*** | PA4263_rplC_at | 760807 | 761106 | 1.39 | -1.925 | **-3.998** | | 50S ribosomal protein L3 | |  |
|  | PA14_08860 | ***rplD*** | PA4262_rplD_at | 767752 | 767928 | -1.19 | **-2.128** | **-3.661** | | 50S ribosomal protein L4 | |  |
|  | PA14_08970 | ***rplE*** | PA4251_rplE_at | 5958489 | 5958704 | -1.48 | -1.485 | -1.968 | | 50S ribosomal protein L5 | |  |
|  | PA14_09000 | ***rplF*** | PA4248_rplF_at | 2240959 | 2241141 | 1.287 | -1.458 | **-2.734** | | 50S ribosomal protein L6 | |  |
|  | PA14_65150 | ***rplI*** | PA4932_rplI_at | 769724 | 769840 | **3.108** | -1.600 | **-2.697** | | 50S ribosomal protein L9 | |  |
|  | PA14_08740 | ***rplJ*** | PA4272_rplJ_at | 761118 | 761939 | 1.54 | -1.938 | **-4.31** | | 50S ribosomal protein L10 | |  |
|  | PA14_08720 | ***rplK*** | PA4274_rplK_at | 759559 | 760194 | 1.127 | -1.292 | -1.639 | | 50S ribosomal protein L11 | |  |
|  | PA14_08750 | ***rplL*** | PA4271_rplL_at | 2469754 | 2469948 | 1.184 | -1.587 | **-3.144** | | 50S ribosomal protein L7/L12 | |  |
|  | PA14_57590 | ***rplM*** | PA4433_rplM_at | 760208 | 760810 | **3.836** | -1.459 | **-2.056** | | 50S ribosomal protein L13 | |  |
|  | PA14_08950 | ***rplN*** | PA4253_rplN_at | 764899 | 765438 | -1.947 | -1.871 | **-4.462** | | 50S ribosomal protein L14 | |  |
|  | PA14_09040 | ***rplO*** | PA4244_rplO_at | 766351 | 766884 | -1.019 | -1.276 | **-4.681** | | 50S ribosomal protein L15 | |  |
|  | PA14_08920 | ***rplP*** | PA4256_rplP_at | 745613 | 745981 | -1.1 | -1.597 | **-2.218** | | 50S ribosomal protein L16 | |  |
|  | PA14_09130 | ***rplQ*** | PA4237_rplQ_at | 5807061 | 5807507 | **-2.265** | -1.449 | **-2.526** | | 50S ribosomal protein L17 | |  |
|  | PA14_09010 | ***rplR*** | PA4247_rplR_at | 745034 | 745534 | 1.077 | -1.356 | **-4.396** | | 50S ribosomal protein L18 | |  |
|  | PA14_16000 | ***rplS*** | PA3742_rplS_at | 743709 | 744140 | **2.434** | -1.582 | **-5.729** | | 50S ribosomal protein L19 | |  |
|  | PA14_28680 | ***rplT*** | PA2741_rplT_at | 764184 | 764552 | **2.581** | -1.345 | -1.482 | | 50S ribosomal protein L20 | |  |
|  | PA14_60460 | ***rplU*** | PA4568_rplU_at | 767932 | 768366 | **4.749** | -1.971 | **-4.209** | | 50S ribosomal protein L21 | |  |
|  | PA14_08900 | ***rplV*** | PA4258_rplV_at | 763287 | 763700 | -1.223 | -1.520 | **-2.798** | | 50S ribosomal protein L22 | |  |
|  | PA14_08870 | ***rplW*** | PA4261_rplW_at | 772440 | 772829 | 1.044 | -1.820 | **-2.281** | | 50S ribosomal protein L23 | |  |
|  | PA14_08960 | ***rplX*** | PA4252_rplX_at | 766895 | 767245 | -1.884 | **-2.258** | **-6.586** | | 50S ribosomal protein L24 | |  |
|  | PA14_61780 | ***rplY*** | PA4671_at | 1362646 | 1362996 | 1.157 | -1.270 | **-4.354** | | 50S ribosomal protein L25 | |  |
|  | PA14_60450 | ***rpmA*** | PA4567_rpmA_at | 2469972 | 2470328 | **5.343** | -1.680 | **-2.28** | | 50S ribosomal protein L27 | |  |
|  | PA14_70190 | ***rpmB*** | PA5316_rpmB_at | 5388778 | 5389089 | **10.679** | 1.072 | -1.649 | | 50S ribosomal protein L28 | |  |
|  | PA14_08930 | ***rpmC*** | PA4255_rpmC_at | 762244 | 762576 | **-2.074** | -1.827 | **-5.616** | | 50S ribosomal protein L29 | |  |
|  | PA14_09030 | ***rpmD*** | PA4245_rpmD_at | 764565 | 764879 | 1.169 | -1.487 | **-6.592** | | 50S ribosomal protein L30 | |  |
|  | PA14_66710 | ***rpmE*** | PA5049_rpmE_at | 5511409 | 5512023 | **6.185** | -1.234 | **-2.033** | | 50S ribosomal protein L31 | |  |
|  | PA14_17700 | ***rpmE2*** | PA3601_at | 5511409 | 5512023 | 1.843 | 1.070 | **3.19** | | 50S ribosomal protein L31 type B | |  |
|  | PA14_25630 | ***rpmF*** | PA2970_rpmF_at | 5388497 | 5388754 | **2.18** | 1.798 | **-2.262** | | 50S ribosomal protein L32 | |  |
|  | PA14_28670 | ***rpmI*** | PA2742_rpmI_at | 6259133 | 6259369 | **2.862** | -1.067 | -1.568 | | 50S ribosomal protein L35 | |  |
|  | PA14_09070 | ***rpmJ*** | PA4242_rpmJ_at | 763700 | 763891 | **-2.674** | -1.046 | **-8.806** | | 50S ribosomal protein L36 | |  |
|  | PA14_17710 | ***rpmJ*** | PA3600_at | 763700 | 763891 | -1.215 | 1.382 | -1.162 | | 50S ribosomal protein L37 | |  |
| **tRNA synthesis** | |  |  |  |  |  |  |  | |  | |  |
|  | PA14_66750 | ***argS*** | PA5051_argS_at | 5961348 | 5963111 | -1.496 | -1.748 | **-3.776** | | arginyl-tRNAsynthetase | |  |
|  | PA14_62730 | ***truB*** | PA4742_truB_at | 5597947 | 5598861 | 1.46 | 1.358 | **-2.709** | | tRNApseudouridine synthase B | |  |
|  | PA14_61790 | ***pth*** | PA4672_at | 5512065 | 5512649 | -1.731 | -1.088 | **-3.5** | | peptidyl-tRNA hydrolase | |  |
|  | PA14_16530 | ***lysS*** | PA3700_lysS_at | 1415600 | 1417105 | -1.871 | -1.213 | **-2.19** | | lysyl-tRNAsynthetase class II | |  |
|  | PA14_15990 | ***trmD*** | PA3743_trmD_at | 1361846 | 1362604 | **6.777** | 1.442 | **-2.027** | | tRNA (guanine-N(1)-)-methyltransferase | |  |
|  | PA14_14440 | ***valS*** | PA3834_valS_at | 1231581 | 1234433 | **-2.394** | -1.899 | **-5.78** | | valyl-tRNAsynthetase | |  |
|  | PA14_58170 | ***gatC*** | PA4482_gatC_at | 5177262 | 5177549 | **4.243** | -1.039 | 1.402 | | aspartyl/glutamyl-tRNAamidotransferase subunit C | |  |
|  | PA14_28650 | ***thrS*** | PA2744_thrS_at | 2467219 | 2469141 | 1.371 | -1.680 | -1.543 | | threonyl-tRNAsynthetase | |  |
|  | PA14_58180 | ***gatA*** | PA4483_gatA_at | 5177565 | 5179019 | -1.165 | -1.540 | **-2.369** | | aspartyl/glutamyl-tRNAamidotransferase subunit A | |  |
|  | PA14_23840 | ***truA*** | PA3114_truA_at | 2072879 | 2073736 | -1.83 | -1.143 | -1.523 | | tRNApseudouridine synthase A | |  |
|  | PA14_00090 | ***glyS*** | PA0008_glyS_at | 10434 | 12488 | **-2.179** | -1.059 | **-2.165** | | glycyl-tRNAsynthetase subunit beta | |  |
|  | PA14_58190 | ***gatB*** | PA4484_gatB_at | 5179126 | 5180571 | **-2.496** | -1.266 | **-2.024** | | aspartyl/glutamyl-tRNAamidotransferase subunit B | |  |
|  | PA14_00190 | ***fmt*** | PA0018_fmt_at | 20090 | 21034 | -1.48 | -1.224 | -1.905 | | methionyl-tRNAformyltransferase | |  |
|  | PA14_65250 | ***hisX*** | PA4939_at | 5814807 | 5815991 | **2.195** | -1.646 | **-2.744** | | histidyl-tRNAsynthetase | |  |
|  | PA14_14890 | ***hisS*** | PA3802_hisS_at | 1264132 | 1265421 | **-2.1** | -1.282 | -1.474 | | histidyl-tRNAsynthetase | |  |
|  | PA14_12230 | ***leuS*** | PA3987_leuS_at | 1055410 | 1058031 | -1.027 | -1.462 | **-4.281** | | leucyl-tRNAsynthetase | |  |
|  | PA14_00100 | ***glyQ*** | PA0009_glyQ_at | 12488 | 13435 | 1.129 | -1.483 | -1.535 | | glycyl-tRNA synthetase subunit alpha | |  |
|  | PA14_28710 | ***pheT*** | PA2739_pheT_at | 2471477 | 2473855 | -1.966 | -1.205 | -1.693 | | phenylalanyl-tRNAsynthetase | |  |
|  | PA14_51820 | ***aspS*** | PA0963_aspS_at | 4601301 | 4603076 | **2.186** | -1.002 | **-2.388** | | aspartatetRNAsynthetase | |  |
|  | PA14_12350 | ***miaB*** | PA3980_at | 1063737 | 1065077 | **2.227** | 1.000 | -1.855 | | (dimethylallyl)adenosine tRNAmethylthiotransferase | |  |
|  | PA14_60370 | ***ileS*** | PA4560_ileS_at | 5379415 | 5382246 | -1.595 | -1.183 | **-2.076** | | isoleucyl-tRNAsynthetase | |  |
|  | PA14_51900 | ***proS*** | PA0956_proS_at | 4608130 | 4609845 | -1.18 | -1.535 | -1.841 | | prolyl-tRNAsynthetase | |  |
|  | PA14_23560 | ***gltX*** | PA3134_gltX_at | 2051388 | 2052872 | 1.227 | -1.712 | -1.293 | | glutamyl-tRNAsynthetase | |  |
|  | PA14_30330 | ***serS*** | PA2612_serS_at | 2627215 | 2628495 | **-2.133** | -1.539 | **-2.302** | | seryl-tRNAsynthetase | |  |
|  | PA14_28690 | ***pheS*** | PA2740_pheS_at | 2470426 | 2471442 | 1.268 | -1.841 | -1.311 | | phenylalanyl-tRNAsynthetase subunit alpha | |  |
|  | PA14_30150 | ***trmU*** | PA2626_trmU_at | 2610796 | 2611923 | 1.885 | -1.043 | -1.386 | | tRNA-specific 2-thiouridylase | |  |
|  | PA14_14600 | ***tgt*** | PA3823_tgt_at | 1245985 | 1247103 | -1.659 | -1.066 | -1.91 | | queuinetRNA-ribosyltransferase | |  |
|  | PA14_73400 | ***thdF*** | PA5567_at | 6533634 | 6535001 | -1.003 | 1.215 | -1.052 | | tRNA modification GTPase | |  |
|  | PA14_05000 | ***micA*** | PA0382_micA_at | 439406 | 440077 | -1.081 | **2.627** | -1.62 | | tRNA (guanine-N(7)-)-methyltransferase | |  |
|  | PA14_67100 | ***yihZ*** | PA5079_at | 5990266 | 5990703 | **-2.374** | -1.059 | -1.488 | | D-tyrosyl-tRNA(Tyr) deacylase | |  |
|  | PA14_48870 | ***ydaO*** | PA1192_at | 4344257 | 4345081 | **2.939** | -1.135 | -1.366 | | C32 tRNAthiolase | |  |
|  | PA14_17440 | ***ygbO*** | PA3626_at | 1495581 | 1496648 | -1.036 | 1.091 | -1.616 | | tRNApseudouridine synthase D | |  |
|  | PA14_57670 | ***trpS*** | PA4439_trpS_at | 5135577 | 5136923 | 1 | -1.549 | -1.299 | | tryptophanyl-tRNAsynthetase | |  |
|  | PA14_30270 | ***aat*** | PA2617_aat_at | 2620775 | 2621455 | -1.58 | -1.400 | -1.479 | | leucyl/phenylalanyl-tRNA--protein transferase | |  |
|  | PA14_62450 | ***trmA*** | PA4720_trmA_at | 5571120 | 5572211 | -1.641 | -1.033 | -1.035 | | tRNA (uracil-5-)-methyltransferase | |  |
|  | PA14_52600 | ***alaS*** | PA0903_alaS_at | 4662149 | 4664773 | -1.053 | -1.222 | -1.495 | | alanyl-tRNAsynthetase | |  |
|  | PA14_41380 | ***glnS*** | PA1794_glnS_at | 3689939 | 3691609 | -1.095 | -1.176 | -1.658 | | glutaminyl-tRNAsynthetase | |  |
|  | PA14_53030 | ***yaeJ*** | PA0868_at | 4701912 | 4702325 | 1.796 | -1.145 | -1.136 | | peptidyl-tRNA hydrolase | |  |
|  | PA14_41360 | ***cysS*** | PA1795_cysS_at | 3688547 | 3689929 | -1.561 | -1.076 | **-2.051** | | cysteinyl-tRNAsynthetase | |  |
|  | PA14_43270 | ***ybbB*** | PA1643_at | 3854107 | 3855216 | **-2.009** | -1.285 | -1.644 | | tRNA 2-selenouridine synthase | |  |
|  | PA14_65320 | ***miaA*** | PA4945_miaA_at | 5820141 | 5821112 | 1.277 | 1.637 | 1.195 | | tRNA delta(2)-isopentenylpyrophosphatetransferase | |  |
|  | PA14_73370 | ***gidA*** | PA5565_gidA_at | 6530561 | 6532453 | 1.743 | 1.228 | -1.083 | | tRNAuridine 5-carboxymethylaminomethyl modification | |  |
|  | PA14_61710 | ***hemA*** | PA4666_hemA_at | 5505528 | 5506796 | **2.691** | 1.039 | 1.043 | | glutamyl-tRNAreductase delta | |  |
|  | PA14_62510 | ***yadB*** | PA4724_at | 5574831 | 5575712 | **3.556** | -1.120 | -1.17 | | glutamyl-Q tRNA(Asp) synthetase | |  |
|  | PA14_08560 | ***tyrZ*** | PA0668_tyrZ_at | 731347 | 732546 | 1.171 | 1.180 | -1.063 | | tyrosyl-tRNAsynthetase | |  |
|  | PA14_14590 | ***queA*** | PA3824_queA_at | 1244929 | 1245972 | **2.968** | -1.442 | -1.249 | | S-adenosylmethionine:tRNAribosyltransferase | |  |
|  | PA14_19050 | ***metG*** | PA3482_metG_at | 1644279 | 1646315 | -1.581 | -1.235 | -1.207 | | methionyl-tRNAsynthetase | |  |
|  | PA14_10420 | ***tyrS*** | PA4138_tyrS_at | 898671 | 899909 | 1.273 | 1.199 | 1.032 | | tyrosyl-tRNAsynthetase | |  |
|  | PA14_27980 | ***yjbN*** | PA2795_at | 2420753 | 2421751 | 1.357 | 1.358 | 1.132 | | tRNA-dihydrouridine synthase A | |  |
| **Initiation factors** | |  |  |  |  |  |  |  | |  | |  |
|  | PA14_30240 | ***infA*** | PA2619_infA_at | 2619908 | 2619690 | **2.084** | -1.297 | 1.012 | | Initiation factor IF-1 | |  |
|  | PA14_64000 | ***yaeJ*** | PA4840_at | 5704564 | 5704193 | 1.296 | **-2.118** | 1.009 | | Initiation factor Sui1 | |  |
|  | PA14_62760 | ***infB*** | PA4744_infB_at | 5599356 | 5601878 | 1.359 | -1.231 | -1.775 | | Initiation factor IF-2 | |  |
|  | PA14_28660 | ***infC*** | PA2743_infC_at | 2469159 | 2469692 | **2.483** | -1.030 | -1.325 | | Iinitiation factor IF-3 | |  |
| **Elongation factors** | | |  |  |  |  |  |  | | |  |  |
|  | PA14_08680 | ***tufB*** | PA4277_at | 741311 | 742504 | 1.046 | 1.428 | 1 | | Elongation factor Tu | |  |
|  | PA14_08830 | ***tufA*** | PA4265_tufA_s_at | 757816 | 759009 | -1.763 | -1.334 | **-3.583** | | Elongation factor Tu | |  |
|  | PA14_08820 | ***fusA1*** | PA4266_fusA1_at | 755665 | 757785 | -1.191 | -1.398 | -1.491 | | Elongation factor G | |  |
|  | PA14_27210 | ***efp*** | PA2851_efp_at | 2364303 | 2364869 | 1.261 | **-2.114** | **-4.361** | | Elongation factor P | |  |
|  | PA14_17070 | ***tsf*** | PA3655_tsf_at | 1463983 | 1464852 | 1.79 | **-2.011** | **-7.15** | | Elongation factor Ts | |  |
|  | PA14_37710 | ***fusA2*** | PA2071_fusA2_at | 3361740 | 3359632 | 1.041 | 1.592 | 1.803 | | Elongation factor G | |  |
|  | PA14_63530 | ***selB*** | PA4807_selB_at | 5665088 | 5663163 | -1.715 | -1.203 | -1.053 | | selenocysteine-specific elongation factor | |  |
| **Release factors** | |  |  |  |  |  |  |  | |  | |  |
|  | PA14_13410 | ***prfC*** | PA3903_prfC_at | 1152100 | 1153683 | 1.331 | -1.414 | -1.922 | | peptide chain release factor 3 | |  |
|  | PA14_61700 | ***prfA*** | PA4665_prfA_at | 5504428 | 5505510 | -1.086 | -1.197 | **-2.589** | | peptide chain release factor 1 | |  |
|  | PA14_72200 |  | PA5470_at | 6432480 | 6431866 | **44.531** | **-2.575** | 1.269 | | peptide chain release factor-like protein | |  |
| **Other translation functions** | | |  |  |  |  |  |  | | |  |  |
|  | PA14_11450 | ***nusB*** | PA4052_nusB_at | 992358 | 992837 | -1.112 | -1.198 | **-3.139** | | Transcript. Antiterm. NusB, Regulates rRNA biosynthesis | |  |
|  | PA14_04860 | ***yhhF*** | PA0370_at | 428681 | 429277 | **-3.276** | -1.194 | -1.678 | | putative methyltransferase, *rRNA* methylation | |  |
|  | PA14_64140 | ***prmA*** | PA4850_prmA_at | 5715852 | 5716736 | 1.823 | -1.261 | **-2.137** | | ribosomal protein L11 methyltransferase | |  |
|  | PA14_62740 | ***rbfA*** | PA4743_rbfA_at | 5598864 | 5599253 | 1.28 | -1.402 | **-3.796** | | ribosome-binding factor A | |  |
|  | PA14_15980 | ***rimM*** | PA3744_rimM_at | 1361312 | 1361839 | **2.788** | 1.288 | **-3.352** | | 16S rRNA-processing protein | |  |
|  | PA14_24650 | ***rmf*** | PA3049_rmf_at | 2154740 | 2154952 | -1.217 | **3.358** | **15.98** | | Ribosome modulation factor | |  |
|  | PA14_73420 | ***rnpA*** | PA5569_rnpA_at | 6537049 | 6537456 | 1.15 | 1.512 | **-2.298** | | ribonuclease P | |  |
|  | PA14_49390 | ***rrmA*** | PA1161_rrmA_at | 4389721 | 4390524 | -1.957 | -1.183 | -1.755 | | rRNAmethyltransferase | |  |
|  | PA14_64190 | ***fis*** | PA4853_fis_at | 5719308 | 5719631 | **3.736** | -1.321 | **-2.782** | | DNA-bind. prot., activator of rRNA and iRNA transcription | |  |
|  | PA14_17100 | ***frr*** | PA3653_frr_at | 1465791 | 1466348 | -1.111 | -1.022 | -1.298 | | ribosome recycling factor | |  |
